# Insights into the spectrum of transtibial prosthetic socket design from expert clinicians and their digital records

**DOI:** 10.3389/fresc.2024.1354069

**Published:** 2024-07-12

**Authors:** A. S. Dickinson, J. W. Steer, C. Rossides, L. E. Diment, F. M. Mbithi, J. L. Bramley, D. Hannett, J. Blinova, Z. Tankard, P. R. Worsley

**Affiliations:** ^1^ Faculty of Engineering & Physical Sciences, University of Southampton, Southampton, United Kingdom; ^2^ Radii Devices Ltd., Bristol, United Kingdom; ^3^ Opcare Ltd., Oxfordshire, United Kingdom; ^4^ Faculty of Environmental and Life Sciences, University of Southampton, Southampton, United Kingdom

**Keywords:** CAD/CAM, PTB, TSB, prosthetic limb design, machine learning, knowledge-based system, expert system

## Abstract

**Background:**

Transtibial prosthetic sockets are often grouped into patella tendon bearing (PTB) or total surface bearing (TSB) designs, but many variations in rectifications are used to apply these principles to an individual's personalised socket. Prosthetists currently have little objective evidence to assist them as they make design choices.

**Aims:**

To compare rectifications made by experienced prosthetists across a range of patient demographics and limb shapes to improve understanding of socket design strategies.

**Methodology:**

163 residual limb surface scans and corresponding CAD/CAM sockets were analysed for 134 randomly selected individuals in a UK prosthetics service. This included 142 PTB and 21 TSB designs. The limb and socket scans were compared to determine the location and size of rectifications. Rectifications were compiled for PTB and TSB designs, and associations between different rectification sizes were assessed using a variety of methods including linear regression, kernel density estimation (KDE) and a Naïve Bayes (NB) classification.

**Results:**

Differences in design features were apparent between PTB and TSB sockets, notably for paratibial carves, gross volume reduction and distal end elongation. However, socket designs varied across a spectrum, with most showing a hybrid of the PTB and TSB principles. Pairwise correlations were observed between the size of some rectifications (e.g., paratibial carves; fibular head build and gross volume reduction). Conversely, the patellar tendon carve depth was not associated significantly with any other rectification, indicating its relative design insensitivity. The Naïve Bayes classifier produced design patterns consistent with expert clinician practice. For example, subtle local rectifications were associated with a large volume reduction (i.e., a TSB-like design), whereas more substantial local rectifications (i.e., a PTB-like design) were associated with a low volume reduction.

**Clinical implications:**

This study demonstrates how we might learn from design records to support education and enhance evidence-based socket design. The method could be used to predict design features for newly presenting patients, based on categorisations of their limb shape and other demographics, implemented alongside expert clinical judgement as smart CAD/CAM design templates.

## 1 Introduction

There are numerous approaches to designing a prosthetic socket to provide a functional body-prosthesis coupling, which transmits tolerable loading to the residual limb during weight-bearing activities. Transtibial prosthetic sockets, for the most common major amputation level, are often grouped by design philosophy. The patella tendon bearing (PTB) approach includes local rectifications to preferentially load relatively tolerant tissues and offload vulnerable sites (1). By contrast, total surface bearing (TSB) sockets are intended to deliver more uniform load distribution and avoid high pressure gradients (2). However, factors like residual limb shape, size, tissue tolerance and desired activity level vary significantly across the heterogeneous population of people with lower limb amputation. In addition, environmental and economic factors need consideration in order to create a comfortable and functional socket, alongside both patient and clinician preference (3).

The International Society for Prosthetics & Orthotics (ISPO) has declared the development of evidence-based socket design to improve fit as a primary objective, in response to calls from prosthetists (4). However, there is limited objective evidence to assist them with design choices for different situations, and often rely on an iterative design process until the prosthesis user finds the limb comfortable (3). The foundational US Veterans’ Affairs Automated Fabrication of Mobility Aids (AFMA) project included analysis of rectification practice (5), and enhanced resolution 3D scan data has led to further such insights recently at the transtibial (6, 7), transfemoral (8) and transradial levels (9). However, but there remains a specific knowledge gap in data to guide the choice of size or combination of individual socket rectification features for a given prosthesis user (10).

There are some clinical indications to support the overall PTB-TSB choice. PTB sockets are generally indicated for longer, more bulbous shaped limbs, and this design principle is commonly used in earlier in prosthetic rehabilitation, especially for people with residual limb pain or oedema (11). TSB sockets are preferred for more mature, stable residual limbs without oedema or excessive soft tissue (12, 13), and are often used for more active individuals, combined with elastomeric liners (14). The PTB rectification pattern design depends on prosthetist judgement and skill, typically achieved through a hands-on plaster method. TSB sockets are also produced by hands-on methods, or by “hands-off” shape capture under hydrostatic pressure, although local shape modification may still be required (15). In practice, inspection of population design data indicates that prosthetists may create hybrid sockets with a spectrum of PTB and TSB features employed to differing degrees (6). However, the relationship between rectification variables remains unclear. Both PTB and TSB sockets can also be produced in the Computer Aided Design and Manufacture (CAD/CAM) approach, and digital design records from CAD/CAM practice present an opportunity to learn from experts.

There is established precedent for these concepts. The use of rectification mapping to describe and communicate socket design was published in 1989 (16), and beside free-hand CAD/CAM, the description of databases of “primitive”, “reference” or “template” sockets with standard rectifications to inform computer aided socket design also dates back to the 1980s (17–21). In the context of much larger adoption of CAD/CAM technologies with higher spatial resolution 3D scans, and evolving principles of socket design, the present study aims to use data-driven methods to conduct an updated study of transtibial socket designs prescribed to a cohort of individuals with lower-limb amputation. This will be achieved by investigating the choice and size of rectifications used by experienced prosthetists, and the combinations of rectification choices they use across a range of design strategies.

## 2 Materials and methods

This was an observational cohort study of transtibial socket design, with approval granted by the University of Southampton ethics and research governance office (ERGO, ref.53279A1). In total 163 sockets, designed in Omega (WillowWood, Ohio, USA) and prescribed to 134 individuals (36F:97M)^1^ were sampled at random from UK clinical service, through a single multi-centre provider (Table 1). The sockets were fitted between November 2018 and November 2022, and the analysed data represented their design prior to any manual adjustment upon fitting. The individuals' demographics and pre-assessed activity level (K-Level) and a post-fitting socket comfort score (SCS) were provided. The researchers were blinded to these data during limb and socket shape data processing, described below.

**Table 1:** Demographics of the recipients of the sampled sockets designs, and distributions of activity (K) level and socket comfort score.

			Sex, *n*		Design, *n*			Age, years		Time since, years			K-Level, 1–4					Socket comfort score, 1–10						
		*n*	F	M	PTB	TSB	PTBSC	Mean	(s.d.)	Med	(range)		1	2	3	4	Mean	5	6	7	8	9	10	Mean
Sex	F	51	51	0	39	9	3	52.9	(16.6)	2.4	0.2	70.3	8	15	28	0	2.4	2	0	6	16	18	7	8.7
	M	111	0	111	95	12	4	59.8	(14.3)	1.0	0.1	53.4	14	46	45	6	2.4	1	3	15	38	29	21	8.4
Design	PTB	135	39	95	135			58.8	(15.4)	0.8	0.1	50.0	20	54	58	3	2.3	2	2	13	47	43	25	8.6
	TSB	21	9	12		21		52.5	(14.0)	10.5	0.3	30.2	2	5	11	3	2.7	1	1	6	6	5	2	8.1
	PTB SC	7	3	4			7	55.6	(16.5)	16.2	6.6	70.3	0	2	5	0	2.8	0	0	2	1	0	1	7.8
Age	19–29	9	4	5	7	1	1	24.2	(3.9)	2.1	0.5	20.9	0	1	7	1	3.0	0	1	0	3	3	1	8.0
	30–39	11	6	5	7	4	0	33.5	(3.5)	2.2	0.2	16.3	1	2	4	4	3.0	1	0	1	6	1	1	7.8
	40–49	28	14	14	24	3	1	47.1	(3.0)	0.8	0.1	50.0	1	8	18	1	2.7	2	0	4	11	6	5	8.0
	50–59	41	11	30	32	7	2	54.7	(2.8)	1.2	0.2	53.4	4	17	20	0	2.4	0	1	8	8	11	12	8.8
	60–69	29	6	23	24	3	2	63.8	(2.5)	0.9	0.2	17.9	1	15	13	0	2.4	0	0	2	11	9	5	8.7
	70–79	39	9	29	35	3	1	74.3	(3.2)	1.8	0.2	70.3	12	15	12	0	2.0	0	1	6	14	15	2	8.3
	>80	6	1	5	5	0	0	86.1	(5.0)	4.0	0.3	17.9	3	3	0	0	2	0	0	0	1	3	2	9.2
Reason for	Dysvascularity	63	11	52	59	3	1	64.8	(12.3)	0.9	0.1	17.9	15	41	6	1	1.9	0	2	8	26	16	10	8.7
	Trauma	47	17	30	32	14	1	51.9	(13.6)	4.6	0.2	46.1	3	10	31	3	2.7	2	1	5	11	16	11	8.7
	Infection	26	10	16	22	2	2	55.3	(18.0)	1.8	0.2	13.6	2	8	16	0	2.5	0	0	2	11	7	3	8.4
	Neuro	10	6	4	10	0	0	53.1	(10.0)	0.6	0.2	6.8	1	1	7	1	2.8	0	0	3	2	4	1	8.2
	Neoplasia	6	4	2	5	1	0	57.7	(22.5)	1.6	0.3	14.0	1	1	3	1	2.7	0	0	0	1	3	2	8.0
	Congenital	3	2	1	2	0	1	29.5	(-)	20.9	0.5	47.8	0	0	3	0	3.0	1	0	0	1	0	0	6.5
Time since amputatn	0–0.25	19	4	15	19	0	0	55.7	(13.3)	0.2	0.1	0.2	3	6	9	1	2.4	0	0	4	9	4	2	8.8
	0.25–0.5	28	8	20	27	1	0	58.5	(14.7)	0.4	0.3	0.5	3	13	11	1	2.4	0	1	4	9	8	5	8.3
	0.5–1	28	6	22	27	1	0	59.0	(15.2)	0.6	0.5	1.0	5	13	9	1	2.2	0	1	3	8	9	5	8.8
	1–2	12	2	10	11	1	0	61.7	(14.9)	1.3	1.0	1.9	2	6	4	0	2.2	0	1	0	5	3	3	8.3
	2–3	12	5	7	12	0	0	52.2	(22.3)	2.4	2.1	3.0	3	3	6	0	2.3	0	0	0	5	4	3	8.8
	3–5	12	6	6	8	4	0	51.1	(13.6)	3.8	3.2	4.8	0	6	6	0	2.5	0	0	3	6	3	0	8.4
	5–10	20	7	12	16	2	1	61.5	(14.9)	7.4	5.3	10.0	4	5	10	0	2.3	1	0	2	5	7	3	8.7
	10–15	13	5	8	7	4	2	62.2	(12.9)	11.5	10.5	14.0	0	4	8	1	2.8	0	0	1	1	5	4	9.0
	15+	17	5	11	8	6	3	57.8	(16.6)	29.0	16.3	70.3	2	2	11	2	2.8	1	0	3	6	5	2	8.0
	Overall	163	51	111	135	21	7	57.7	(15.4)	1.2	0.1	70.3	22	61	74	6	2.4	3	3	21	54	48	28	8.5

**Figure 1:**
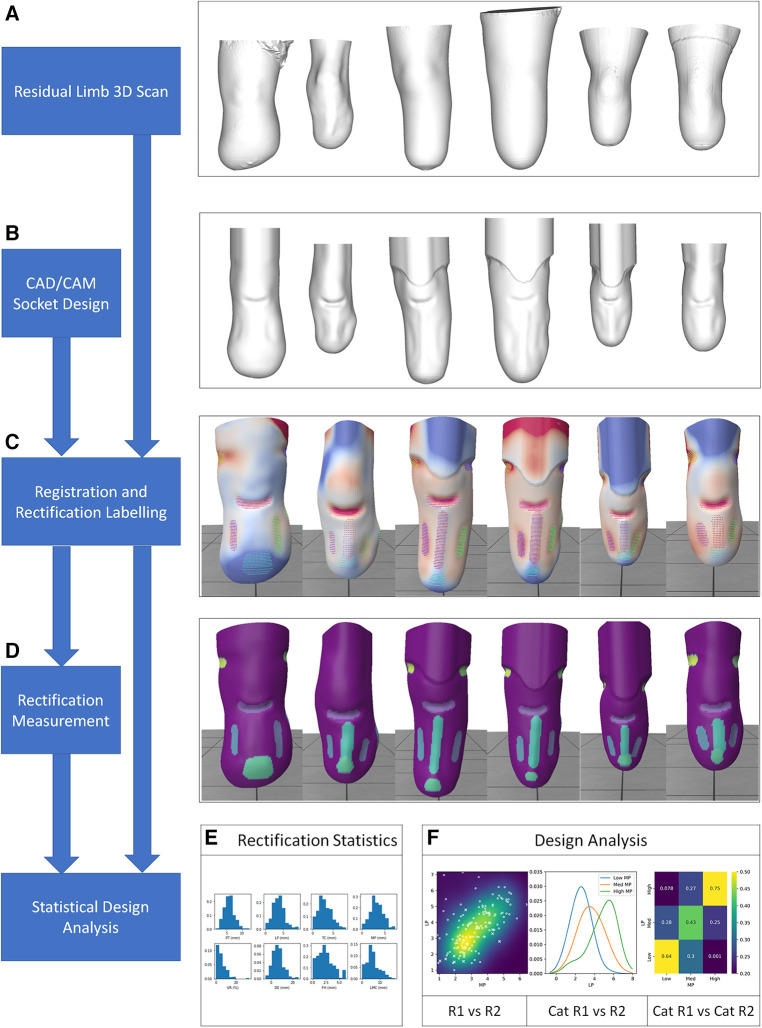
Data processing from 3D scan of limb and CAD/CAM socket design, extracted rectification design feature locations and sizes, expressed as design variables, and categorised.

Two surface meshes were obtained for each participant, representing a 3D scan of the residuum and the corresponding mould design file shape used to produce the socket (Figures 1A,B). The residuum and rectified socket scan pairs were aligned using the ampscan open source toolbox (22), first coarsely using a calculated principal axis and manually-picked mid patella and distal tibia landmarks, and then more precisely using an automatic, iterative closest point (ICP) process operating on the anterior, sub-patellar portion of the shape. Finally all aligned pairs were inspected by two experienced observers (AD, JS) and small manual adjustments were made where necessary. The shapes were then registered to one-another using ampscan to describe each socket's design as a rectification map (Figure 1C). Clusters of scan mesh vertices representing individual rectifications were identified manually by two experienced observers (AD, JS) (Figure 1D), and within each cluster the rectification “size” was obtained, as the depth of carve or height of build-up from limb to socket surfaces (Figure 1E). The 98th percentile deviation across the vertices in each rectification cluster was used instead of the maximum, to avoid any noise arising from individual vertices. This method was used to describe “design features” of local rectifications at the patellar tendon (PT, carve), fibula head (FH, build), medial and lateral paratibial areas (MP, LP, carves), the tibial crest (TC, build), distal end elongation (DE, build), and between the lateral and medial supracondylar regions (LMC, carves). Further, a gross socket sizing design variable was calculated as the volume reduction (VR) by finding the mean of cross-sectional area differences between the limb and socket at 10 sections between the mid-patella tendon and distal end of the tibia.

The rectification data were analysed in three stages:

• To characterise the study population and ensure representativeness and coverage, exploratory data analysis inspected the distribution of sex, age, reason for amputation, time since amputation, socket comfort score (SCS) and K-Levels, and prescribed socket design. The population's age distribution was normally distributed so parametric descriptive statistics were used (mean and standard deviation, s.d.). The time since amputation was not normally distributed, and so the median and range were reported.
• To understand general socket design trends, the sizes of PT, FH, LP, MP, TC, DE, SC, and VR rectifications were analysed. Differences in the extent to which the rectifications were used in sockets designed using PTB and TSB approaches was compared using the non-parametric Mann-Whitney *U*-Test (rectification size distributions were not normally distributed). Bonferroni *post hoc* correction to reduce the risk of Type-I errors arising from multiple comparisons.

Finally, associations between the separate rectifications' sizes were assessed, to inspect more subtle trends in expert prosthetists' rectification strategy (Figure 1F):

• First, to evaluate simple correlation between the sizes of pairs of rectifications, Spearman Rank regression was calculated. This method can detect linear correlations but cannot rule out more complex non-linear associations and is highly influenced by outliers. Therefore:
• The probabilistic methods Kernel Density Estimation (KDE) and Gaussian Naïve Bayes classification (23) were applied to further investigate the diversity and frequency of different design approaches, and search for causal relationships between rectifications. These analytical methods estimated the probability of a prosthetist's choice of one rectification size following a prior decision of another rectification size. This enabled interrogation of the expert prosthetist's training datasets to find the probabilities of selecting, for example, a low, medium, or high build at the Fibular Head given a high carve at the Patellar Tendon. These categories were identified by splitting the fitted KDE function at the 33rd and 67th percentiles.

## 3 Results

### 3.1 Exploratory data analysis

Exploratory Data Analysis revealed differences in demographics, activity assessment and socket comfort across the population (Table 1). The studied socket designs were prescribed to a population with a widely distributed age (*n* = 134, mean 58.6 years, range 19.6–94.1 years), and were delivered over a range of times since amputation or limb absence (*n* = 163, median 1.2 years, range 0.14–70.3 years). The sockets were prescribed for a range of reasons for limb absence, which included dysvascularity (39%), trauma (29%) and infection (16%). Twenty-one were designed to a TSB principle (13%), 7 as PTB supracondylar sockets (4%) and the rest were “standard” PTBs. The dataset was sparse for people with congenital limb absence (3 individuals), people aged over 80 years (6 individuals), and only included adults.

Compared to the whole cohort, people with amputations due to trauma were observed to have higher activity (mean K level 2.7 vs. 2.4), were longer post-amputation (median 4.6 years vs. 1.2 years), and more likely to use TSB sockets (14/47, 30% vs. 21/163, 13%). People with dysvascularity-related amputations were older (mean 65 years vs. 58 years), had lower activity than the population averages (mean K level 1.9), and had their amputations more recently (median 0.9 years). TSBs were prescribed to people with longer-established amputations than PTBs (median 10.5 years vs. 0.8 years).

### 3.2 Descriptive statistical analysis of expert socket design practice by rectification

**Figure 2:**
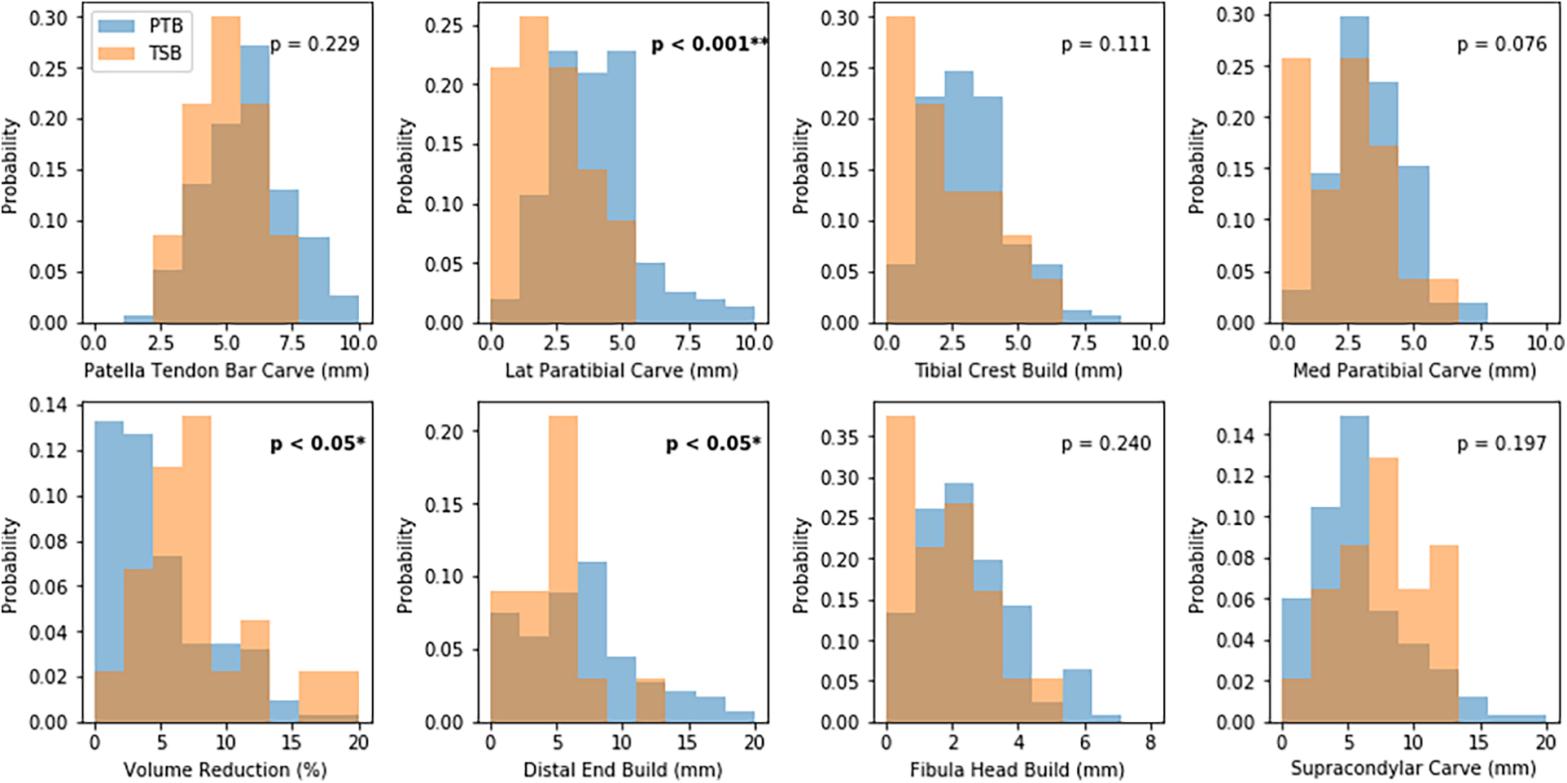
Distributions of rectification sizes for sockets described as PTB and TSB designs. “Build” denotes material is added, and “Carve” denotes material is removed.

Several design features were used across sockets described during design as PTB or TSB (Figure 2). Local rectifications were typically larger in PTB sockets than TSBs, and this difference was statistically significant for the DE elongation build (*p* < 0.05), LP carve (*p* < 0.001) and approached significance for MP carve (*p* = 0.076). Conversely, the gross volume reduction (VR) was significantly larger for TSBs (*p* < 0.05). However, a considerable overlap was observed between all rectification distributions, and notably the PT carve and FH build rectification sizes were similar across both groups.

**Figure 3:**
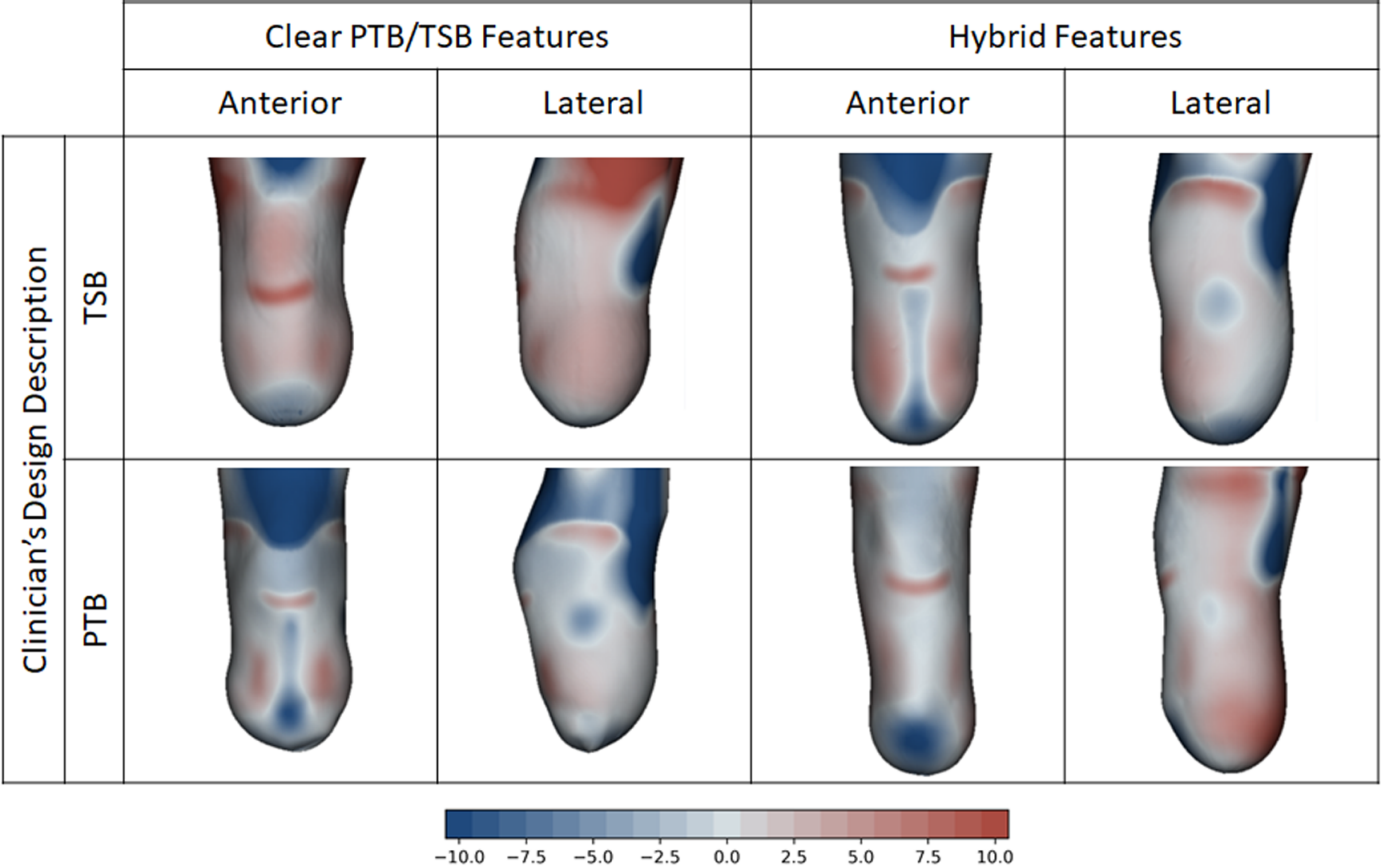
Example socket designs to PTB and TSB intent, plotted on the residual limb shape. Some training dataset designs had clear PTB or TSB intent, and others lay on a hybrid spectrum between PTB and TSB. Colour key indicates rectification design map in mm. Positive (red) represents carve, and negative (blue) represents build-up.

The training dataset was observed to contain sockets that were clearly recognisable as PTB or TSB designs, and others which appeared to contain more hybrid features (Figure 3). Therefore, instead of analysing the socket population in discrete groups, design was evaluated using rectification sizes as continuous variables.

Multiple linear correlation (Table 2) revealed several associations between the sizes of rectification pairs. There was a significant positive correlation between LP and MP rectifications (*ρ* = 0.66, p < 0.001), which are features that are often performed together. Moderate negative correlations were observed between the off-loading build at the tibial crest (TC) and both MP and LP paratibial carves (*ρ* = −0.40, *p* < 0.001 and *ρ* = −0.35, *p* < 0.001, respectively), features which are often performed together and are more pronounced in nominally PTB sockets. A significant positive correlation was observed between the off-loading build at the fibular head (FH) and the gross volume reduction (VR), (*ρ* = 0.38, *p* < 0.001). This is also expected: a build is used to offload the FH bony prominence in PTB sockets whereas a line-to-line fit is preserved here in TSB sockets, which typically use greater VR to achieve more uniform load transfer. Weaker negative correlations were also observed between builds at the distal end elongation (DE) and at the fibular head (FH) (*ρ* = −0.32, *p* < 0.001). However, the patellar tendon (PT) rectification depth did not correlate significantly with any other rectification, indicating its relative design independence.

**Table 2:** Spearman rank correlations (*ρ*) between rectification groups.

	PT	MP	LP	FH	DE	VR	TC	LMC
PT	–							
MP	0.18	–						
LP	0.18	0.66**	–					
FH	0.11	−0.17	−0.26**	–				
DE	−0.15	0.05	0.11	−0.32**	–			
VR	−0.17	−0.13	−0.25**	0.38**	−0.19	–		
TC	0.10	−0.40**	−0.35**	0.37**	−0.18	0.21	–	
LMC	−0.20	0.16	−0.07	0.01	−0.09	0.22	−0.14	–

* denotes significance at p < 0.05, **at p < 0.001. Positive correlations occur where both rectifications are builds or carves, and negative where one is a build and the other is a carve.

### 3.3 Probabilistic analysis of socket design practice

The raw dataset carve and build rectification sizes were split into low-, mid- and high-sized categories with limits at the population 33rd and 67th percentiles. These were further reduced to exemplar single values of low- middle- and high-sized rectifications at the 10th, 50th and 90th percentiles (Table 3).

**Table 3:** Categorised rectification sizes extracted from the KDE function fitted to the training dataset of 163 socket designs.

		Category		
Rectification		Low (10th %le)	Mid (50th %le)	High (90th %le)
Patellar Tendon, mm	(carve)	4.1	5.8	7.4
Fibular Head, mm	(build)	1.0	2.1	3.8
Medial Paratibial, mm	(carve)	1.9	3.2	4.7
Lateral Paratibial, mm	(carve)	2.1	3.6	5.1
Tibial Crest, mm	(build)	1.4	2.7	4.4
Distal End, mm	(build)	1.5	5.8	10
Lateral-Medial Condyles, mm	(carve)	3.0	5.5	9.3
Volume Reduction, %		1.5	4.3	9.9

**Figure 4:**
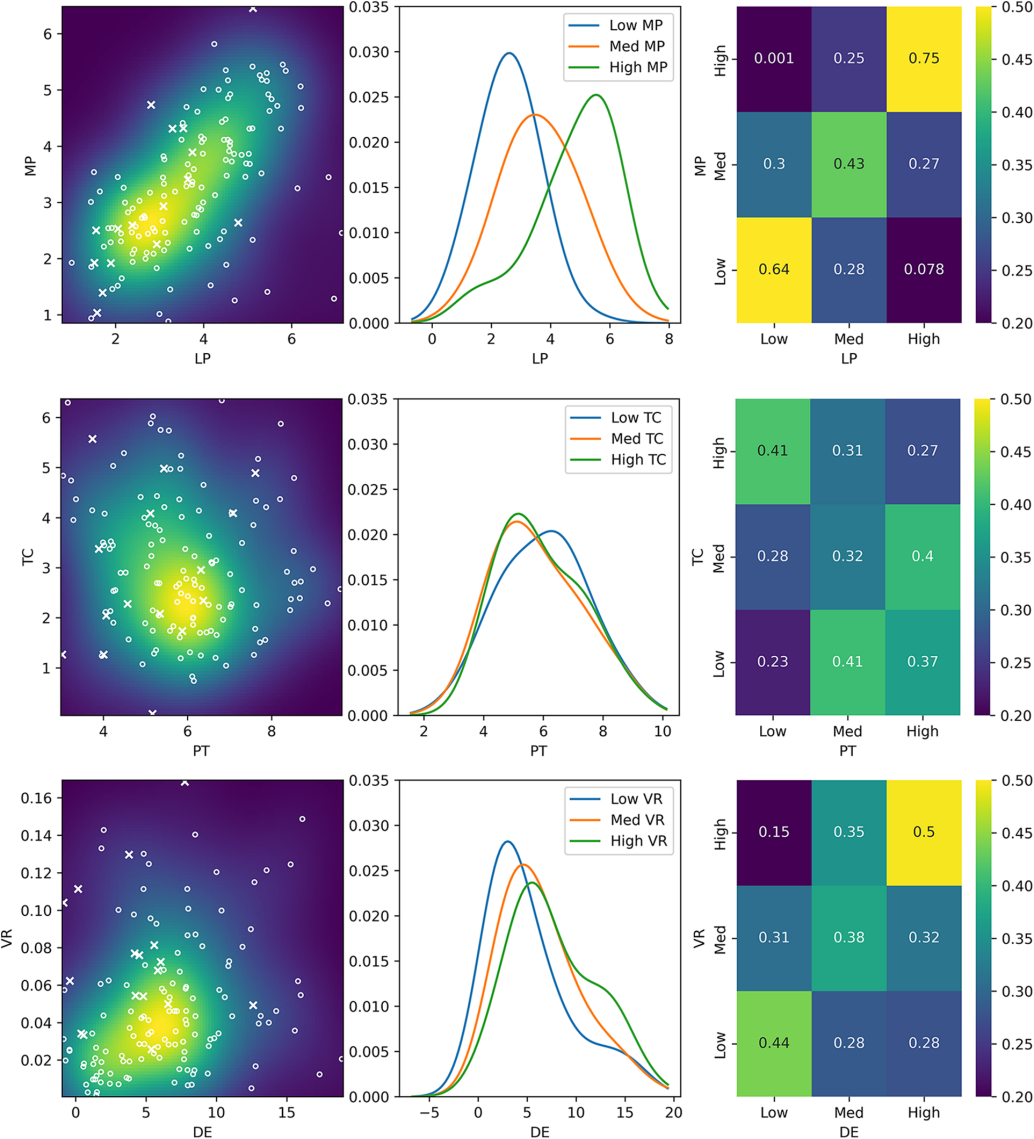
Three methods of assessing association between the sizes of three example pairs of rectifications. First, a scatter plot (left) of rectification sizes shows common combinations of rectification sizes, where each point represents one of the 163 training sockets. Both variables are continuous. The probability of combinations calculated by Kernel Density Estimation (KDE) is superimposed as a colour map. Circles represent nominally PTB sockets, and crosses are nominally TSB sockets. Three slices through the dataset are then used (centre) to define low, medium and high values of one rectification. For these categories, the corresponding probability density function of the other rectification is plotted. Finally, the Gaussian Naïve Bayes (NB) classifier is used to show the probability that a prosthetist would choose combinations of low, medium and high sizes of each rectification having previously chosen the size of another rectification (right). Results are shown for a highly associated pair (LP and MP, top), an un-associated pair (PT and TC, middle) and a pair which contains different association options (DE and VC, bottom).

Simple associations existed for some rectification pairs, for example a strong correspondence between the size of medial and lateral paratibial carves (Figure 4 top). This was evidenced by a strong linear correlation, and a low probability from the KDE and NB analyses that a high medial paratibial carve would be used in combination with a low lateral paratibial carve, and vice versa (<10%).

Other rectification pairs were not associated. In particular, the choice of patellar tendon carve depth did not strongly influence any other rectification choice, which was evidenced by weak correlations and similar probabilities in the KDE and NB analyses (minimum 23% and maximum 41%, where random choice between sizes is 33%; Figure 4 middle).

However, the associations between some rectification pairs were more complex, and distinctly different clinical strategies were apparent, notably for the gross volume reduction which is often one of the first rectification choices made during the design process. Following the choice of gross volume reduction to apply, clinicians made different choices of whether to elongate the distal end to accommodate displaced soft tissue (Figure 4 bottom). For example, in the case of a low volume reduction, there was some causal link to the choice of distal end elongation (low 44% vs. high 28%), which may reflect a choice to offload the distal tip. However, for a high volume reduction, the causal link was much stronger (low 15% vs. high 50%), supporting the requirement of more space at the distal end to accommodate the soft tissues when they are highly compressed.

**Figure 5:**
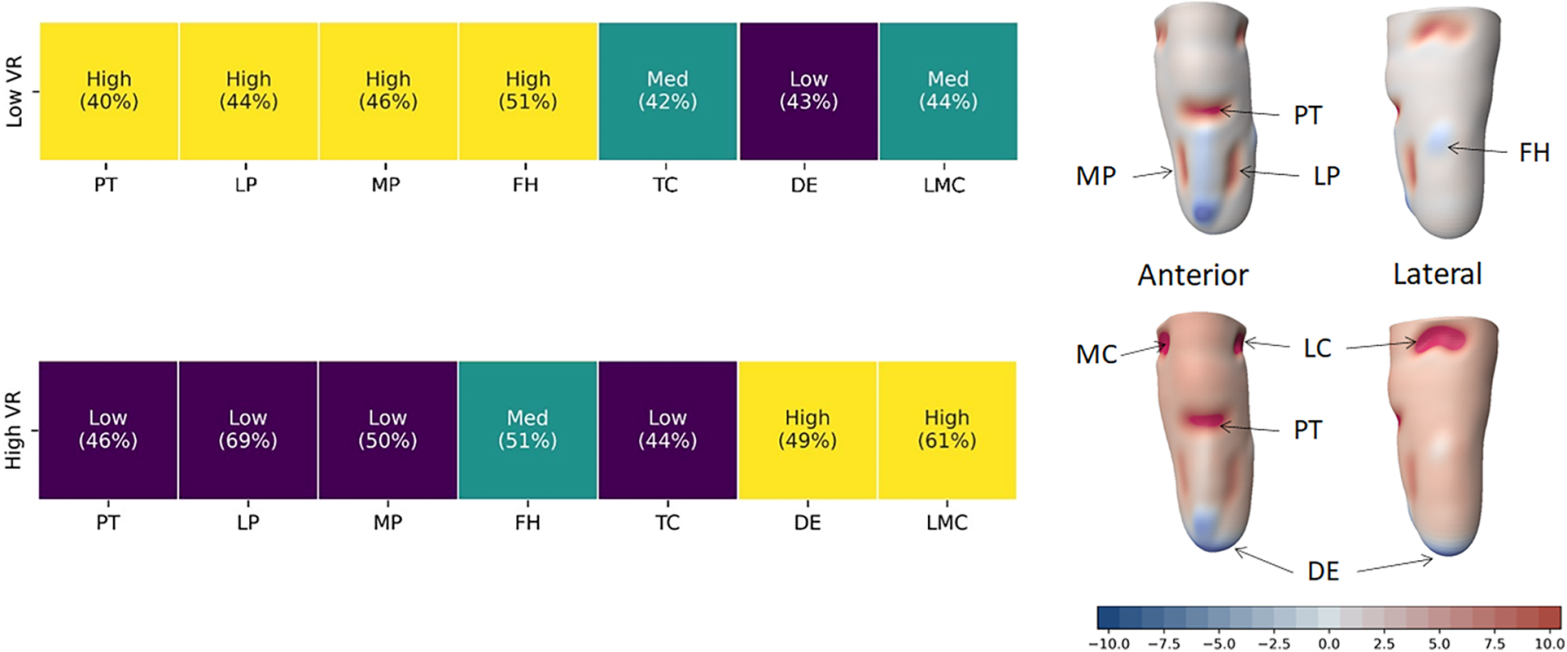
By pre-defining the size of volume reduction across the socket, the Naïve Bayes classifier was used to provide probabilities of the clinician choice of size of the other rectifications across the training dataset. With 3 categories, a probability of 33.3% would represent no preference. This shows clear groups of more PTB-biased socket design associated with a decision to perform low volume reduction (top row) and more TSB-biased features associated with a large-sized volume reduction (bottom row). In rectification map, red represents a carve or volume reduction in the socket design, blue represents build, and white is a close match to the limb shape.

Finally, to demonstrate an example use case of these insights from expert clinical practice, the Naïve Bayes classifier was used to create example socket designs with the highest probability to result from an initial clinical decision of a high or low volume reduction. The resulting rectifications were superimposed upon the mean residual limb shape from the training population of 3D scans [Figure 5 (6),]. For sockets with a low degree of volume reduction, prosthetists were most likely to use more pronounced carves at the patellar tendon and paratibials, a high FH offload, a mid-sized tibial crest offload and a mid-to-low distal end elongation, collectively representing more PTB-like design features (Figure 5 top). Conversely, for sockets with a high volume reduction, prosthetists used small carves at the patellar tendon, paratibials and tibial crest, a closer-fitting FH profile, and a large distal end elongation, features commonly used together in more TSB-like sockets, along with lateral-medial carves above the knee condyles (Figure 5 bottom).

## 4 Discussion

This study set out to enhance our objective understanding of prosthetic socket design. We assessed the spectrum of transtibial socket features in a randomly sampled UK population, by identifying and measuring the selection and size of rectifications used by experienced prosthetists, and associations between these choices.

The study presents quantitative data that express how CAD/CAM sockets designed by expert prosthetists to PTB and TSB approaches do not form clearly separate groups, but lie on a spectrum. Local rectifications were typically smaller, and the volume reduction was typically larger for the TSB group compared to the PTB group. However, across the study population there was considerable overlap between all rectification sizes for PTB and TSB designs, which supports the biomechanical theory that rectifications work together, and therefore associations between chosen rectification sizes were inspected.

Strong linear correlations were observed between the sizes of rectifications which typically feature in combination, in PTB designs. The PT carve depth was not associated with any other rectification, indicating its relative design insensitivity. Similarly, supracondylar carves varied independently from all rectifications, consistent with these being more “optional” design features, consistent with their role in suspension rather than the transfer of stance loads. It was also noteworthy that despite finding no simple correlation between elongation at the residuum's distal end and volume reduction, the variables were associated. For example, a large volume reduction was rarely used without an associated distal elongation to accommodate the displaced soft tissue. Such logical but more complex associations between rectification sizes were not detected by linear regression but were revealed by applying probabilistic approaches.

Rectification practice insights like these might be used in combination with variables of residuum size and shape extracted from a new limb scan, to identify the most likely combination of rectifications that prosthetists have used to design sockets for similar cases in the past. The resulting rectifications could be presented to prosthetists as “templates”, to support at the beginning of their design process, incorporating the understanding of the interdependence of these local design decisions. There are considerable evidence, economic, operational and mindset factors involved in implementing digital technologies in prosthetics clinic workflows (24), and many considerations for socket design beyond a person's residual limb size and shape. For this reason, we would never recommend that such analysis of past rectifications is used to automate socket design, and an expert prosthetist should always remain responsible; they know their client best. The rationale is the same as in CAD/CAM, where a 3D surface scan alone will not identify highly person-specific sites of sensitivity or vulnerable tissue such as wounds, scars, grafts and bony prominences or heterotopic ossification. Such cases may explain the outliers visible in Figure 4 (left). Although the great majority of sockets had less than 10% volume reduction and less than 10 mm distal end elongation, the presence of outliers illustrates and reinforces the importance of expert clinical intervention, to meet individual needs for sockets with design features lying outside the normal size range.

Beyond direct residuum-based factors influencing socket design choices, prosthetists will include practical, service-delivery and usability considerations. The cost of current PTB and TSB options is reported to be equivalent in the short term, with PTB costing 40% less initially but requiring a greater number of clinic visits with their associated time and travel costs, over three times as long, to achieve equivalent clinical performance (25). Part of the cost, function and comfort benefits of TSB sockets may be attributed to corresponding vacuum assisted suspension and silicone or elastomer liners, although these are reported to produce more perspiration and require manual skill in donning, which may be more difficult for older individuals and people with impaired manual dexterity (26).

The study uses a retrospective analysis of sockets from 3D scanned residual limb surface and CAD/CAM socket design data alone. As mentioned above, prosthetists also consider soft tissue composition and sensitive or vulnerable sites in their design, based on palpation, but this information was unavailable for the present study. The study's training data also considered only CAD/CAM PTB and TSB sockets, and different findings might be obtained if sockets produced using conventional plaster-based processes were digitised and studied by the same methods. Furthermore, the study also does not provide information on the negative effects of poor design, or undesirable rectification choices, because all sockets included in the training population were relatively comfortable; 80% of the population had an SCS > 7. Other rectification features may also be relevant beyond the size or depth used in this study, such as the rectification zone area, shape and location, but were not considered in this study.

Furthermore, though this study employs a larger population than previously published modelling and socket analysis studies, its generalisability is inevitably limited. The study's exploratory data analysis revealed trends which agreed with previously published research, and the use of PTB and TSB approaches matched clinical guidelines. Comfort level trends agreed with clinical assessments for conventional PTB and hydrocast TSB sockets (higher for PTB, and increasing with time since amputation) (27), and trends in TSB socket users indicated higher activity and higher satisfaction amongst young, active users (28, 29). The exploratory data analysis also showed some heterogeneity in sex, age and reason for amputation which was representative of the UK NHS population (30), but there may be preference for design to different styles in different locations. External validity beyond the present setting may also be limited because other patient groups in different ecogeographic groupings or ethnicities will present different anatomic, pathology and surgical variations, which may require different clinical management. Prosthetists might use the presented methods to perform detailed analysis of their own prior practice or for similar patients seen by colleagues or peers in a practice or region (19), or as in the current exemplar dataset this method might be used to investigate trends across a broader population. The presented methods are built upon open-source software tools and can be applied to other historic design records, but the results should not in isolation be interpreted as recommendations for clinical practice. Finally, while the study was designed to provide detailed observational descriptions of socket design, it does not provide a direct mechanistic explanation of these designs' load transfer. The results are best interpreted in conjunction with mechanical and clinical tests which attempt to understand these mechanisms (31, 32) and link them to clinical effectiveness in terms of function and quality of life (3, 11, 33), towards the study's stated aim of enhancing our community's evidence-based support for socket design.

## 5 Conclusion and clinical implications

This study set out to derive objective understanding from population-based socket design records, towards supporting clinicians to reduce the iterative socket design in prosthetic limb provision. Sockets were shown to vary in a spectrum, instead of separate clusters of more pure PTB or TSB approaches, so future clinical studies should look at the design paradigm with continuous variables instead of discrete groups. This understanding might be implemented clinically in the form of initial modified geometry, or as a list of modification sizes which could be applied in a predefined workflow in conventional CAD/CAM software, or in CAD/CAM templates. As described previously, such templates should be selected and adapted to the patient by certified prosthetists (5, 6, 8, 18, 19, 21), and as suggested by Boone et al. in the ShapeMaker system (19) they could also be updated, learning from a prosthetist's individual technique, or data might continue to be pooled for more general insights. Such templates would not substitute clinical training but might free the prosthetist to focus more of their time on the higher value-added, patient-facing part of their practice.

Ultimately the intention of this paper's methodology is to provide a tool for prosthetists to understand their range of decision making and learn more about alternative methods to achieve the same result. Knowledge derived using these methods may also enhance how clinicians share best practice for complex cases, and how less experienced prosthetists and trainees learn from analysing the work of highly skilled prosthetists. The results also provide insights to support engineers in conducting physical testing and biomechanical simulations that represent real-world clinical practice.

## Data Availability

The data analyzed in this study is subject to the following licenses/restrictions: Raw datasets analysed during the study are part of individuals' healthcare data. Ethical approval was granted for the study to access them under Secondary Data Analysis, but the raw data cannot be made publicly available for reasons of individual privacy. Processed data behind the figures is however made publicly available. The dataset supporting the conclusions of this article is available in the University of Southampton repository, https://doi.org/10.5258/SOTON/D2896.

## Footnotes

^1^counts which add up to less than the total indicate a missing metadata point. For example there was no record of sex for one person, reason for limb absence for one person, or time since amputation for 3 people
